# A collection of FAIR Dutch Freedom of Information Act documents

**DOI:** 10.1038/s41597-025-05052-2

**Published:** 2025-05-15

**Authors:** Ruben van Heusden, Maik Larooij, Jaap Kamps, Maarten Marx

**Affiliations:** 1 University of Amsterdam, Informatics Institute, IRLab, Amsterdam, Netherlands; 2 University of Amsterdam, Faculty of Humanities, Amsterdam, Netherlands

**Keywords:** Scientific data, Computer science

## Abstract

When Dutch citizens want to gain insights into the decision-making process of their government, they can file a so-called Freedom of Information Act request, requesting information on specific topics. The resulting documents (released publicly) have the potential to be a valuable resource for the research community, both in the domain of computer science, as well as the social- and political sciences. However, the current publication landscape is very scattered, with many organizations publishing on their own websites, with little to no coordination on document structure, (meta)data quality, and without a standardized metadata format. In this paper we present a collection of these documents published as FAIR data. The dataset contains just over two million pages, collected by scraping supplier websites, after which document metadata standardization was performed, and checks were carried out to ensure text- and metadata quality. The document text- and layout, their metadata, and where available links to the original PDF files, are all available through the DANS data repository, including usage instructions and examples.

## Background & Summary

Like over a hundred countries worldwide, the Netherlands has Freedom of Information Act (FOIA) legislation, requiring governmental institutions to release documents related to their decision-making process to the public, either passively or proactively^1^. Generally speaking, the aim of this kind of legislation is to increase government transparency, allowing citizens to examine the decision-making process, addressing possible inconsistencies, and therefore facilitating the proper functioning of the democratic process^2,3^.

Apart from the benefits for the democratic process, a collection of these FOIA documents can also be valuable for different disciplines within the research community. However, the current creation- and publication process of these kinds of documents in the Netherlands makes it very difficult to use this potentially valuable resource in research, as it often fails to adhere to the FAIR data guidelines outlined by Wilkinson *et al*.^4,5^. Currently, each agency is responsible for publishing their own records, usually on their own platform or website, with little to no coordination between agencies on what kinds of metadata are included, and no standards on text quality and machine-readability. The result of this is that conducting large-scale research across different agencies is complicated, as the data needs to be collected from multiple sources, metadata needs to be standardized, and there is no guarantee that the quality of the data is sufficient for usage in research.

In an attempt to mitigate the aforementioned problems, and to make this valuable resource of FOIA documents available as FAIR data, we created the publicly accessible *Woogle* dataset. The Dutch FOIA is abbreviated as Woo and we ensure that Woo-data is easily searchable, hence the name.

By creating a uniform set of metadata in a standardized format (e.g., ISO-dates for all date related events, a fixed set of document types), we facilitate both the *findability* and the *interoperability* of the document collection. The *accessibility* of the documents is handled by not only having the documents be freely available, but also by having persistent metadata, even if the source of the document no longer exists. Finally, the *re-useability* of the dataset is addressed by having high-quality machine-readable text available for the documents in the collection.

In 2022, the Dutch FOIA legislation was broadened significantly, including not only *passive* requests of documents (a request sent to a specific government agency), but also requiring the *active* release of documents. This means that governing bodies are obliged to release these documents on a public website satisfying quite strict technical criteria, which resemble the FAIR principles.

In this paper, we limit ourselves to the portion of the Woogle dataset containing passive requests, as the collection of documents resulting from active release is currently far from complete, and internationally rather unique. However, the process of collection and FAIRification of these documents is identical to that of the passively released documents.

Having such a broad collection of documents from a large number of organizations provides a wealth of opportunities for multiple purposes. For instance, searching for protected animal species like wolf, certain toads or deer yields hundreds of hits and shows when, where and in what context governing bodies deal with this topic. Besides facilitating search across different agencies, the collection also provides a wealth of information for researchers in both the computer science and political science domains, allowing for example the large-scale study of government policies on public housing or refugees, or studies on the popularity of certain keywords or topics in the requests for information. For the field of Natural Language Processing (NLP), it not only opens up the possibility of several forms of text analysis, but also allows for the training of new language models that are better tailored to this specific domain. This can in turn be used to provide improved interaction with these documents, with for example simplification or automatic summarization of the information, making this type of information accessible to a larger number of people (which after all is their main purpose).

Although the source language of the Woogle dataset is Dutch, the usefulness of the dataset is not limited to Dutch research efforts only, and the dataset can also be of value to the international research community. The presence of high-quality metadata means that it is possible to filter documents based on specific criteria, such as a certain time period, or a certain organization, and then further process those files. One can for example select only files from the Dutch parliament during the COVID pandemic, and then use automatic translation to translate the documents into another language for further analysis. The advantage of this corpus is thus not only the information itself, but also its structured format, which avoids having to manually retrieve this data, which can be especially troublesome for someone not familiar with the structure of the Dutch governing body, as well as avoiding having to translate the entire corpus, where now only translating a subset would be sufficient.

Throughout Europe and the United States, there are several similar initiatives and collections regarding released FOIA documents, both on the national and regional levels. Examples of regional collections and initiatives can be found in Hamburg, where a search engine for government documents was launched in 2014 (https://transparenz.hamburg.de/ueber-un), and Brussels, where a similar effort to digitize FOIA documents was undertaken (https://transparencia.be). An example of a national database of FOIA documents is the Norwegian Electronic Public Records (OEP) platform, which contains roughly 70 million documents from municipalities, ministries, and several other governmental agencies (https://einnsyn.no/statistikk/generell). The platform allows users to search the collection and maintains an agenda of political meetings for which documents are available in the repository. In a similar vein, the European Parliament has also created an online portal for accessing previously released documents, and provides means for citizens to request information that is not (yet) available online (https://www.europarl.europa.eu/RegistreWeb/home/welcome.htm?).

There are also several related initiatives regarding the publication of FOIA documents in the United States, the two most prominent examples being FOIArchive from the History-Lab project (http://history-lab.org) and the collection of documents released as part of the FOIA Project from the TRAC research center at Syracuse University (https://foiaproject.org). The History-Lab collection consists of eight subcorpora, totaling roughly three million documents ranging from 1861 to 2013, and is among the largest collections of de-classified government documents in the United States. Apart from the ability to search the document collection, the corpus also contains automatically extracted topics and Named Entity annotations. The FOIA Project dataset from the TRAC initiative consists of roughly 9,000 documents relating to decisions made on the withholding or releasing of data by the federal government, including formal decisions as well as the results of lawsuits regarding publication. Apart from merely having these FOIA documents available as online information, the dissemination of the data also facilities the development of more complex analyses of different aspects of the data. An example of this is the COVID-19 Archive Prototype(https://covid19-prototype.history-lab.org), which is a collection of emails from an American medical expert, obtained through FOIA requests, and extracted from the History-Lab collection. The data was processed so that it can be filtered not only by content, but also by automatically extracted topics. A part of the History-Lab collection was also used for the development and testing of a system for automatically extracting important events in government documents using Machine Learning techniques^6^.

The rest of the paper is organized as follows. The Methods section starts with an explanation of the used model of FOIA dossiers, and its operationalization used in the dataset. We continue with an overview of the dataset, the collection process, and the FAIRification process. The Records section contains a detailed description of the different types of information included in the dataset, and the Technical Validation section elaborates on the validation performed on the quality of the dataset. We conclude with some final notes on best practices for using the dataset in the Usage Notes and the usage of third-party software in the Code Availability section.

## Methods

### Dataset Structure

In the dataset, we follow the structure of a typical FOIA request, where each received request is considered a *dossier*, containing two mandatory files (the original request and a decision letter, detailing whether the request is granted or denied, and for what reasons) and, depending on whether or not the request is granted, an inventory list of released documents, and the released documents themselves (often partially redacted).

Both the dossier and the documents contained within it come with a standard set of metadata. A dossier typically has the following metadata associated with it.

- The date of the original request and the date of the decision letter
- The actual request (information need) and the decision (granted or denied)
- The government body that took the decision and released the documents and the (url of the) source

Key metadata of documents are whether they are fully or partially made public (that is, whether they contain redacted text), the legal reasons applied to refuse publication of certain parts of the text, and the kind of document released.

The advantage of this document structure is that it is somewhat universal, at least from the perspective of having a dossier structure associated with a request. The same model has also been applied to a collection of Estonian FOIA documents, and it resembles the model of a structured literature search, with distinct groupings of documents into clusters^7,8^.

### Dataset Overview

The dataset presented in this paper is a static version of the documents in the Woogle search engine on *04-11-2024*. The portion of the Woogle dataset relating to the passively released FOIA documents contains 13, 529 dossiers, 121, 175 documents and 2, 122, 100 pages. The dataset contains around 378 million words, with 4.2 million unique words. The dataset contains documents released between 2001 and 2024.

**Fig. 1:**
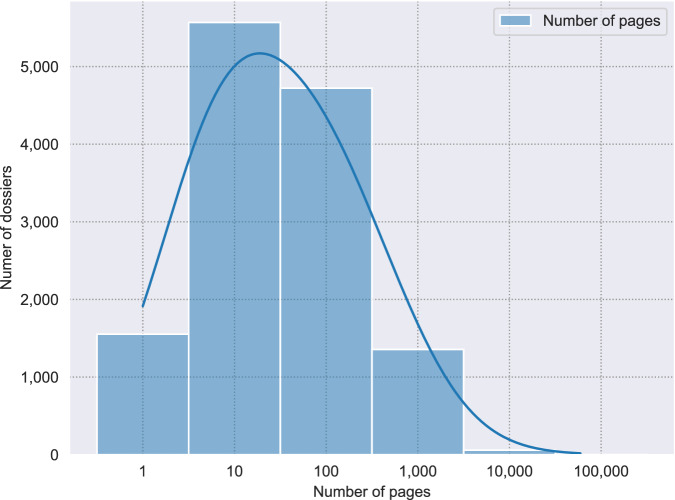
Distribution of the number of pages per dossier, with log-x scale (N = 13,529, *μ* = 158).

Figure 1 gives an overview of the distribution of the number of pages per dossier, and Table 1 gives an overview of the distribution of pages and words over the four documents types discussed above.

**Table 1:** The number of pages and number of words for the four different document types in the dataset.

Document Type	Number of Pages	Number of Words
Released Documents	1,825,461	322 million
Decision Letter	245,349	47 million
Original Request	25,554	4 million
Inventory List	25,736	4.5 million
**Total**	2,122,100	377.5 million

**Fig. 2:**
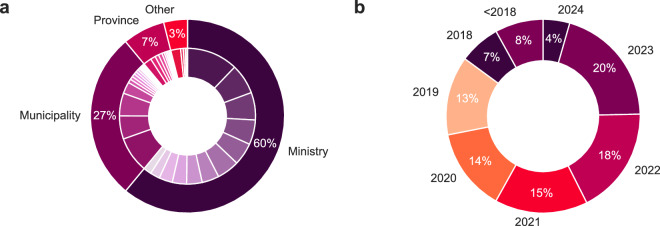
Distribution of dossiers of 59 suppliers (inner ring), grouped by the type of organization (outer ring) (**a**), Distribution of the number of dossiers released each year (from 2001 to 2024) (**b**).

Figure 2a,b provide an overview of the types of suppliers of dossiers, as well as the number of released dossiers through time. The majority of the dossiers originate from either ministries or municipalities, and the majority of dossiers have been released over the last five years. The inner ring shows that the production of dossiers within types of governing bodies is rather balanced and not Pareto distributed.

### Dataset Collection

All the FOIA dossiers released by Dutch ministries are published on a central open government platform (https://open.overheid.nl/). On this platform, filters can be applied to download only the passively published documents (*Beslissing op Wob-/Woo-verzoek*in Dutch), and the results can be filtered to include only documents from ministries. Several municipalities are subscribed to the *OpenPub* platform, where they publish their FOIA documents, which can be accessed through an API.

Most governing bodies which are not local governments use somewhat standardized centrally provided publication software, where the information can be extracted in a way similar to the way in which the ministry documents are extracted. For other government bodies, custom scraping software was built to extract the information from the respective agency website.

A more detailed description of the scrapers and their source code is also available (in Dutch) on GitHub (https://github.com/wooverheid/WoogleDocumentatie) as well as in the *DatasetCollection* notebook present in the dataset repository. According to Dutch law, documents released under the Freedom of Information Act are by default subject to the CC-BY license, allowing re-use, if appropriate credit is given.

### FAIRification

We strive to collect all basic metadata described above in the FOIA dossier model in our scraping process. If it is consistently made available, we collect it and normalize the data (converting different dating conventions to ISO format, classifying different requester types). The type of the document (decision, request, released document and inventory list) is not always explicitly listed, and thus has to be extracted using heuristics based on the filename and file-type. We attempt to obtain machine-readable text from the documents using the *pdftotext* software (https://www.xpdfreader.com/pdftotext-man.html) if a text layer is available in the PDF document. If this is not the case, or if the quality of the original text is too poor, Optical Character Recognition (OCR) software is used to extract text from the images. Tesseract version 5 with Sauvola binarization (https://tesseract-ocr.github.io) is used for OCR. Detecting whether a document needs to be OCRed is also a heuristic process performed with the *OCRmyPDF* software (https://ocrmypdf.readthedocs.io/en/latest/). We store both the original text (obtained from the PDF using *pdftotext*), and the text obtained by Tesseract.

#### Text Quality

For assessing the overall text quality of a document, it is meaningful to have a single score indicating the machine-readability of that document. We refer to this score as the *FAIRIscore*, which rates the overall readability of a document according to five classes from A to E, where E is the lowest and A is the highest. The explanation of the classes is given below. The name, the 5 FAIRIscore values, the colors and the assignment of values to items have been inspired by the Nutri-Score^9^. To determine whether a page is scanned in, we use the *MuPDF* software (https://mupdf.com) to determine whether or not a page contains an image that covers (almost all of) the page, indicating a scan. Note that there is no standard procedure to test whether a PDF document is “digital born”, meaning that it has been created for instance by an export-as-PDF command in Word. Therefore we must resort to these heuristic methods.

- **A** None of the pages in the document has a page-covering image, and the original text of the document contains at least 100 characters on every page.
- **B** The document contains some (but not only) page-covering images, but the average Jaccard similarity between the original text and the OCRed text over all pages in the document is larger than . 9. Also true if the document contains no page-covering images, but some pages have less then 100 characters in the original text.
- **C** The document contains some (but not only) page-covering images, and the Jaccard similarity between the original text and the OCRed text over all pages of the document is less than . 9 but higher than the mean similarity over all documents.
- **D** The document contains some (but not only) page-covering images, and the Jaccard similarity between the original text and the OCRed text over all pages of the document is less than the mean similarity over all documents.
- **E** The document contains no machine readable letters in the original text, and all pages in the document consist of page-covering images.

**Fig. 3:**
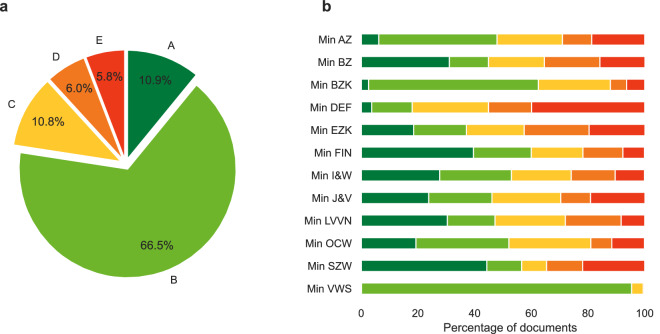
Distribution of the FAIRIscore for all FOIA documents released by ministries (**a**) Distribution of the FAIRIscore for all FOIA documents released by ministries, grouped by ministry (**b**).

Figure 3a,b give an overview of the FAIRIscore distribution for all the documents released by ministries. In general, the quality of the documents released by the ministries is reasonable, with more than 75 percent of the released documents having a score of either A or B. There is quite some variability between the different ministries however, as shown in Fig. 3b, where for some ministries the number of documents of lesser quality (score C or below) being more than half of the total number of documents. We note that we deliberately have chosen not to include any automatically testable PDF-accessibility criteria as described in PDF/UA and WCAG 2.1 because the vast majority of the Dutch Woo PDF documents does not adhere to these.

#### Page Stream Segmentation (PSS)

During the construction of the dataset, we found that in more than 90% of the cases where documents were being scanned in, multiple documents were scanned in consecutively, and ended up being saved as one large PDF file. Naturally this is undesirable, as we want the individual documents to be findable, and not only have them as part of a very large PDF file, without document-specific metadata. The task of reconstructing the original documents from these *streams* of pages is known in the literature as Page Stream Segmentation (PSS), and several approaches based on Machine Learning techniques have been proposed for this task. Most recent approaches to this task use methods based on neural networks to classify individual pages on whether or not they start a new documents, using both textual- and visual features^10–12^. The segmentation of the documents in the Woogle dataset is performed using the text-based method from Wiedeman & Heyer^10^. The reason behind using this approach is that previous research has shown that this leads to models that are more robust to input from different sources (as opposed to image-based models), which is important for the Woogle dataset, as there is a large number of different document suppliers^13^.

**Fig. 4:**
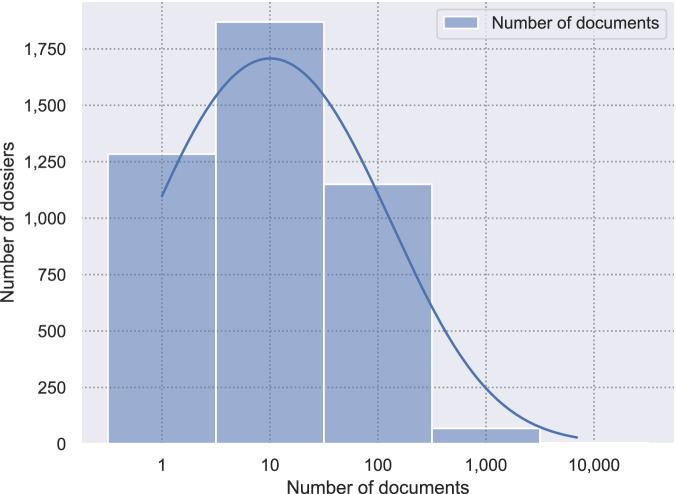
Distribution of the number of documents of the *Released Documents* class per dossier after applying PSS (N = 4,377, *μ* = 44, log-x scale).

Figure 4 shows the distribution of the number of documents of the *Released Documents* class in a dossier, after the application of the segmentation algorithm. We focused on this class as it is most prone to containing document scans, given that the documents might come from various different sources, whereas decision letters are often a single document created via text-processing software. After applying PSS to the *Released Documents*, the number of documents increased from 37, 570 to 192, 663. The median document length decreased from 26 to 5 pages, and the longest document is now 2, 755 pages long, compared to 4, 401 pages before segmentation. The algorithm was evaluated in previous work, where it achieved an F1 score of . 78 measured on two datasets^13^.

#### Redacted Text Detection

As some of the information in the documents is confidential or privacy-sensitive, organizations will redact the confidential information from the document before releasing it to the public. However, as there is no clear standard for redaction practices or redaction software, a plethora of different methods is used, from automatic redaction using regular expressions to manual redaction by pen. Although some of these methods retain the original metadata of the document (such as the embedded text), other methods, such as the printing, redacting and scanning techniques, do not preserve this information. As a result, text-to-speech software might not know how to properly deal with these redactions, and thus a PDF containing text redaction might be unintelligible when read aloud by a computer. To provide an overview of the extent of redaction in the dataset, we measure the amount of redacted text using Machine Learning techniques. The used algorithm for detecting redacted text is described in van Heusden *et al*.^14^.

**Fig. 5:**
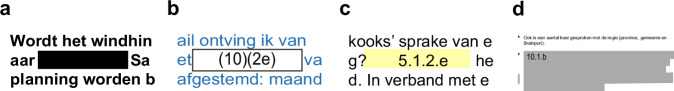
Examples of different types of redaction in the dataset. The codes in the redactions are not type dependent. The color redaction can appear in different colors. The gray redactions can appear in different shades of gray.

**Fig. 6:**
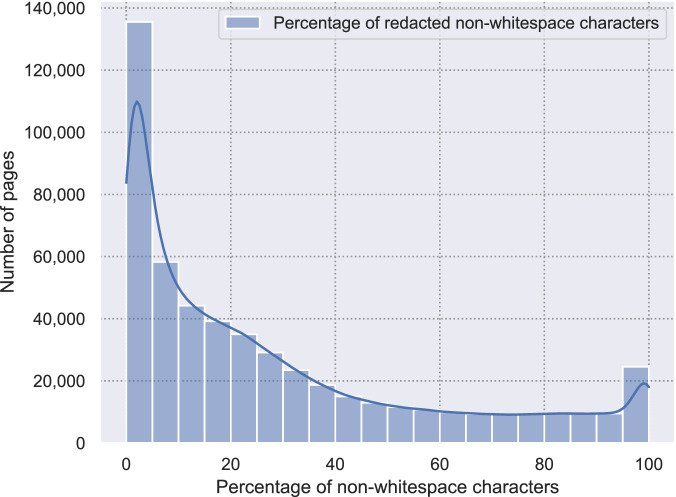
Distribution of the percentage of non-whitespace characters on a page that are, for pages that contain at least one redaction (N = 523,603, median = 18%).

For the 1, 172, 713 pages for which the analysis was performed, 523, 603 of them (around 45 percent) contained at least a single redaction, with a total of 4, 359, 857 redactions on these pages (around 8 redactions per page). On pages that contained at least a single redaction, the median percentage of redacted characters was around 18, and around two percent of these pages were completely redacted. Figure 5 shows examples of the different styles of redactions in our corpus. Figure 6 shows the distribution of the portion of the characters on a page that is redacted. The performance of the algorithm is evaluated in previous work, with an F1 score of . 77 on a manually annotated test collection^14^.

## Data Records

The dataset is available at the DANS Data Station for Social Sciences and Humanities^15^. The data presented in this paper is stored in three separate files in the *dataset* folder, namely the *woo_dossiers.csv.gz*, *woo_documents.csv.gz* and *woo_bodytext.csv.gz* files, containing the dossier, document and page metadata respectively. Apart from this, the actively released documents are also stored in the dataset as extra material in the *EXTRA-active-release* folder. The dataset is available under the CC BY 4.0 license (https://creativecommons.org/licenses/by/4.0/deed.en), allowing users to use and modify the data for their own purposes (giving appropriate credit).

The dossiers, documents and pages all have separate sets of metadata associated with their records, which are stored in a separate file for each of these types. The dataframes containing the records all adhere to the Boyce-Codd Normal Form (BCNF) for databases. In these dataframes, the *dc_identifier* provides a unique ID for each of the rows in the dataframe, and for the document and bodytext dataframes the IDs of the dossier and documents respectively are included to allow the joining of the different dataframes. The precise contents of the dataframes is detailed in Tables 2, 3 and 4. The majority of these attributes are extracted automatically during the scraping process, and some attributes are added after processing of the document, as those discussed in the previous section. In the tables we only report on the missing values if they occur in at least 5 percent of values for that attribute.

**Table 2:** Overview of the fields in the *woo_dossiers.csv.gz* archive, where every row describes a dossier.

Field	type	Description	Fraction of NaNs
*dc_date_year*	date	Year when the dossier was released	—
*dc_description*	string	Short description of the dossier contents	0.18
*dc_identifier*	string	Unique dossier identifier	—
*dc_publisher*	string	Code of the dossier publisher	—
*dc_publisher_name*	string	Full name of the publisher	—
*dc_source*	string	URL pointing to the source of the dossier	0.07
*dc_title*	string	Title of the dossier	—
*dc_type*	string	Type code of dossier	—
*dc_type_description*	string	Human-readable version of the *dc_type*	—
*foi_decisionDate*	date	Date of decision on request	0.17
*foi_decisionText*	string	Explanation of decision	0.98
*foi_isAdjourned*	string	Whether or not the request is adjourned	0.98
*foi_publishedDate*	date	Date of the publication of the dossier	0.17
*foi_requestDate*	date	Date of original request	0.88
*foi_requestText*	string	Text of the original request	0.92
*foi_requester*	string	requester type (organization, individual)	0.99
*foi_retrievedDate*	date	Date on which the dossier was retrieved	—
*foi_valuation*	string	Decision on a request	0.98

The *dc_identifier* attribute is the primary key.

**Table 3:** Overview of the fields in the *woo_documents.csv.gz* archive, where every row describes a document.

Field	Type	Description	Fraction of NaNs
*dc_format*	string	Format of the document (MIME type)	0.38
*dc_identifier*	string	Unique document identifier	—
*dc_source*	string	Source of the document (document URL)	0.40
*dc_title*	string	Title of the document	0.49
*dc_type*	string	Type of document (As described in the Methods section)	—
*foi_dossierId*	string	Identifier of the dossier to which the document belongs	—
*foi_fairiscoreVersions*	string	FAIRIscore of the document	—
*foi_fileName*	string	Filename of the document	—
*foi_nrPages*	int	Number of pages in the document	—

The *dc_identifier* attribute is the primary key, and the *foi_dossierId* is the foreign key.

**Table 4:** Overview of the fields in the *woo_bodytext.csv.gz* archive, where every row describes a page.

Field	Type	Description	Fraction of NaNs
*foi_bodyText*	string	Text of the page extracted using *pdftotext*	0.23
*foi_bodyTextOCR*	string	Text of the page extracted using Tesseract	—
*foi_bodyTextJaccard*	float	Jaccard similarity between *foi_bodyText* and *foi_bodytextOCR*	—
*foi_charArea*	int	Number of pixels on the page belonging to a character	0.45
*foi_contourArea*	int	Number of pixels on the page belonging to a redaction	0.45
*foi_documentId*	string	Identifier of the document to which the page belongs	—
*foi_hasOCR*	bool	Whether there is OCR text available	—
*foi_imageArea*	float	Percentage of a page that is covered by an image	—
*foi_imageCoversFullPage*	bool	Whether a single image completely covers an image	—
*foi_isFirstPageOfNewDoc*	bool	Whether a page is the start of a new document (PSS analysis)	0.43
*foi_nrRedactedRegions*	int	Number of individual redacted regions	0.45
*foi_pageNumber*	int	The number of the page within a document	—
*foi_percentageCharAreaRedacted*	float	Percentage of the characters on a page that is redacted	0.45
*foi_percentageTextAreaRedacted*	float	Percentage of the total text area that is redacted (including whitespace)	0.45
*foi_redacted*	bool	Whether there is redaction present	0.45
*foi_textArea*	int	Number of pixels on a page which belong to text	0.45

The *foi_documentId* is the foreign key, and the *foi_documentId* combined with the *foi_pageNumber* attribute is the (composite) primary key.

In addition to the datafields mentioned in Table 3, the dataframe with document metadata contains an additional 9 metadata fields, all relating to metadata extracted from PDF files using the *pdfinfo* tool (https://www.xpdfreader.com/pdfinfo-man.html). As these fields have data values for a very small portion of the documents (between one and two percent of all documents), and will generally not be available for scanned documents, we did not include them in the description of the dataframe. As previously mentioned, the *foi_bodyText* attribute contains the text of a page (if this was present, otherwise the text is extracted using OCR), together with layout information of the text. For most use cases, such as constructing a search engine, this information will be sufficient, however there might be instances in which the original PDF has to be accessed. The *dc_source* attribute contains the URL from which the document was originally retrieved. In some cases, this URL has become invalid, for example because the structure of a website was changed. In this case the *dc_identifier* attribute can be used to retrieve the original document from the the project server using https://doi.wooverheid.nl/?doi=combined with the dc_identifier of that particular record, for example https://doi.wooverheid.nl/?doi=nl.gm0148.2k.2020.396.doc.1 (a notebook with more information about this process is present in the dataset).

## Technical Validation

When evaluating the quality of a dataset, there are several key dimensions that should be considered. Two dimensions that are commonly considered are *Consistency*/*Validity* and *Completeness*^16,17^. Here consistency or validity refers to whether the values in a dataset are valid for that type of data (right datatype, in the right range), and completeness refers to how much of all data is captured in the dataset.

We further decompose the completeness of our dataset into *extrinsic* and *intrinsic* completeness, where extrinsic completeness refers to how much of the dossiers that exist are present in the dataset, and intrinsic completeness refers to the amount of missing values in the dataset.

In our case, the extrinsic completeness of the dataset is hard to measure, as the variety of different suppliers and websites makes it difficult to judge how much of the suppliers we have dossiers for. For the municipalities we have dossiers of 31 municipalities, out of all 342 municipalities in the Netherlands. For the ministries we have dossiers for all of them, as these are published on a single platform, and can be scraped with a single script. Our main focus is to ensure that, for the publishers that we have scrapers for, we collect all of the dossiers that are published on their websites. We do this by, apart from having scrapers to update the dataset daily, periodically re-scraping the websites to catch any publications that might have had incorrect dates.

For all the dossiers that we collect, we attempt to retrieve the metadata listed in the tables in the Records section. If this data is available, we parse the data, converting it into the required format. This includes converting dates to ISO format, standardizing dossier- and document types into the pre-defined sets of allowed values (e.g., a document type must belong to the four discussed types) or translating fields to binary attributes. To ensure the validity of the data, this parsing includes validation of the input, i.e. ensuring that parsed dates and numerical values are within the expected range, and that binary attributes only contains boolean values.

Reporting on the intrinsic completeness of the dataset, we briefly comment on all fields with at least 5% missing values. We start with the missing values in Table 2. The *dc_source* attribute points to the original source (URL) of the document, and in cases where PDF files were submitted directly to us by government agencies, this URL is not available.

The *dc_description*, *foi_decisionText*, *foi_isAdjourned*, *foi_requestText*, *foi_requestDate*, *foi_requester* and *foi_valuation* attributes are all fields that are published by some suppliers, but not by all, and also not (easily) extractable from the dossier contents. The *foi_publishedDate* and *foi_decisionDate* are available for most suppliers, but when agencies submit directly to us, they are not strictly required, and thus missing for some dossiers. For the attributes in Table 3, the *dc_format*, *dc_source* and *dc_title* attributes have missing values for more than 5 percent of their attributes. As with the dossiers, this information is not always supplied by suppliers, and automatic extraction is complicated and not very reliable. For the pages in Table 4, the *foi_bodyText* attribute has missing values as for about a quarter of the data there is simply no machine-readable text in the document, possibly a result of scanning. All the other missing values in the table are due to the fact that the PSS- and redacted text detection algorithms are rather expensive to run, and were only included in the processing pipeline in mid 2023.

## Usage Notes

Apart from the dataframes with the dataset, the repository also contains several notebooks that provide guidance on loading the data and working with the dataset. The *Woogledataset.ipynb* notebook is the most important, as it contains instructions on how to load in the datasets, and shows several examples on how to work with the data, as well as containing the code used to generate the numbers and plots in this paper. The dataset described in this paper is a static version of the contents of the Woogle search engine, which is continuously updated with new documents. This static dataset has version number 4, which can be used to access the version of the dataset associated with this paper. Since the number of documents collected is constantly increasing, new versions of the dataset will be made available through DANS in the future.

## Data Availability

For the collection of the dataset, web-scrapers coded in Python were used, together with code to periodically fetch data from APIs, the code is available on Github (https://github.com/wooverheid/WoogleDocumentatie). The code used to perform the PSS segmentation and redacted text detection is available on GitHub (https://github.com/irlabamsterdam/OpenPSSbenchmark, https://github.com/irlabamsterdam/TPDLTextRedaction). The usage of other third-party software used in the creation of the dataset has been detailed in the Methods section.
